# Changes in pediatric pneumonia incidence and pathogen spectrum before, during, and after the COVID-19 pandemic: an eight-year inpatient data analysis from a tertiary hospital in Fujian, China

**DOI:** 10.1186/s12887-026-07008-w

**Published:** 2026-05-21

**Authors:** Zhenhua Xia, Huiqun Zhang, Shuzheng Xu, Xiaoling Lin, Fei He, Xiaoyan Zhang

**Affiliations:** 1 Department of Pediatrics, The First Affiliated Hospital of Fujian Medical University, Fuzhou, Fujian China; 2 Department of Epidemiology and Health Statistics, School of Public Health, Fujian Medical University, Fuzhou, Fujian China; 3 Allergy Center, The First Affiliated Hospital of Fujian Medical University, Fuzhou, Fujian China; 4 School of Basic Medical Sciences, Fujian Medical University, Fuzhou, Fujian China; 5 Department of Otolaryngology Head and Neck Surgery, The First Affiliated Hospital of Fujian Medical University, Fuzhou, Fujian China; 6 Department of Otolaryngology Head and Neck Surgery, National Regional Medical Center, Binhai Campus of the First Affiliated Hospital, Fujian Medical University, Fuzhou, Fujian China; 7 Fujian Institute of Otorhinolaryngology, The First Affiliated Hospital of Fujian Medical University, Fuzhou, Fujian China; 8 Fujian Clinical Research Center for Difficult Otorhinolaryngologic Diseases, The First Affiliated Hospital of Fujian Medical University, Fuzhou, Fujian China

**Keywords:** Community-acquired pneumonia in children, COVID-19, Incidence, Pathogen spectrum, Co-infection

## Abstract

**Objective:**

To analyze the incidence and etiologic distribution of pediatric community-acquired pneumonia (CAP) in Fujian Province, China, across the periods before, during, and after the COVID-19 pandemic from 2017 to 2024.

**Methods:**

We retrospectively analyzed 2,878 hospitalized pediatric CAP cases from 2017 to 2024. Cases were grouped into pre-pandemic (2017–2019), mid-pandemic (2020–2022), and post-pandemic (2023–2024) periods, and compared epidemiological and etiological characteristics.

**Results:**

The overall pathogen positivity rate among children with pneumonia was 82.73%. In the pre-pandemic, mid-pandemic, and post-pandemic phases, the positivity rates were 88.22%, 68.90%, and 83.71%, respectively, with a statistically significant difference (*P* < 0.001). The predominant pathogens were *Mycoplasma pneumoniae* (MP, 36.31%), influenza virus (Flu, 20.29%), and respiratory syncytial virus (RSV, 2.26%). During the pandemic period, the overall infection rate, MP infection rate, and co-infection rate decreased significantly, whereas the proportion of RSV infections increased. In the post-pandemic period, MP infection rates increased markedly, along with a significant rise in both MP mono-infection and MP + Flu co-infection. The overall co-infection rate was 19.04%. Among co-infections, the most frequent combinations were MP+FluB and MP+FluB+FluA. Patients with co-infection involving Flu and MP had a higher proportion of severe pneumonia, compared to those with pure viral co-infection (FluB+FluA).

**Conclusion:**

This single-center study suggests that COVID-19 control measures were associated with temporal shifts in the pathogen spectrum of childhood pneumonia. Notably, infection rates decreased markedly during the pandemic, but rebounded post-pandemic, characterized by a surge in MP and co-infections alongside a decline in RSV. These findings highlight the need for sustained surveillance to optimize prevention and treatment strategies in the post-pandemic era.

## Introduction

Community-acquired pneumonia (CAP) in children remains one of the leading causes of hospitalization and mortality worldwide. Its etiology is influenced by multiple factors, including host immune status, environmental exposures, and public health interventions [1–3]. Since the emergence of coronavirus disease 2019 (COVID-19), the implementation of stringent non-pharmaceutical interventions (NPIs) in many countries was associated with changes in the epidemiological patterns of respiratory infectious diseases [4–6]. Prior to the pandemic, *Mycoplasma pneumoniae* (MP) was reported to be the predominant causative agent of CAP in children in China [7]. However, during the peak of the COVID-19 outbreak, the number of hospitalized CAP cases declined markedly, accompanied by a significant reduction in MP infection rates and notable shifts in the relative proportions of other pathogens, including bacteria and respiratory syncytial virus (RSV) [8, 9].

Currently, most studies investigating the impact of COVID-19 on the epidemiological features of childhood CAP have focused on short-term changes during the pandemic period, with limited reports on longitudinal analyses covering the entire pre-pandemic, mid-pandemic, and post-pandemic phases (2017–2024). In particular, systematic investigations specific to the Fujian region remain scarce. Therefore, this study aimed to conduct a retrospective analysis of pediatric pneumonia cases admitted to the First Affiliated Hospital of Fujian Medical University between 2017 and 2024, systematically comparing temporal changes in infection rates and pathogen distributions across different pandemic stages. The findings are intended to provide a scientific foundation for optimizing prevention and control strategies for childhood respiratory tract infections in the post-pandemic era.

## Subjects and methods

### Study population

This was a retrospective study of hospitalized children with pneumonia admitted to the Department of Pediatrics, the First Affiliated Hospital of Fujian Medical University, between January 2017 and December 2024. A total of 3,022 children were initially screened. Among them, 56 patients did not meet the diagnostic criteria for CAP, leaving 2,966 children assessed for eligibility with a diagnosis of CAP. Inclusion criteria [10]: (fulfillment of at least one item from ①–④, combined with criterion ⑤): ① Worsening of pre-existing respiratory manifestations or new onset of productive cough, cough, purulent sputum, or chest pain; ② Fever; ③ Abnormal white blood cell count indicated by routine blood test (WBC < 4 × 10⁹/L or > 10 × 10⁹/L); ④ Presence of moist rales on pulmonary auscultation or signs of lung consolidation; ⑤ Imaging evidence of patchy or segmental infiltrative shadows or interstitial changes on chest radiography/computed tomography, which may be accompanied by pleural effusion.

All enrolled cases were diagnosed with CAP. Sputum, pharyngeal swabs, and serum specimens were obtained within 24 h of admission and subjected to bacterial culture, nucleic acid testing, and antigen/antibody detection.

**Fig. 1:**
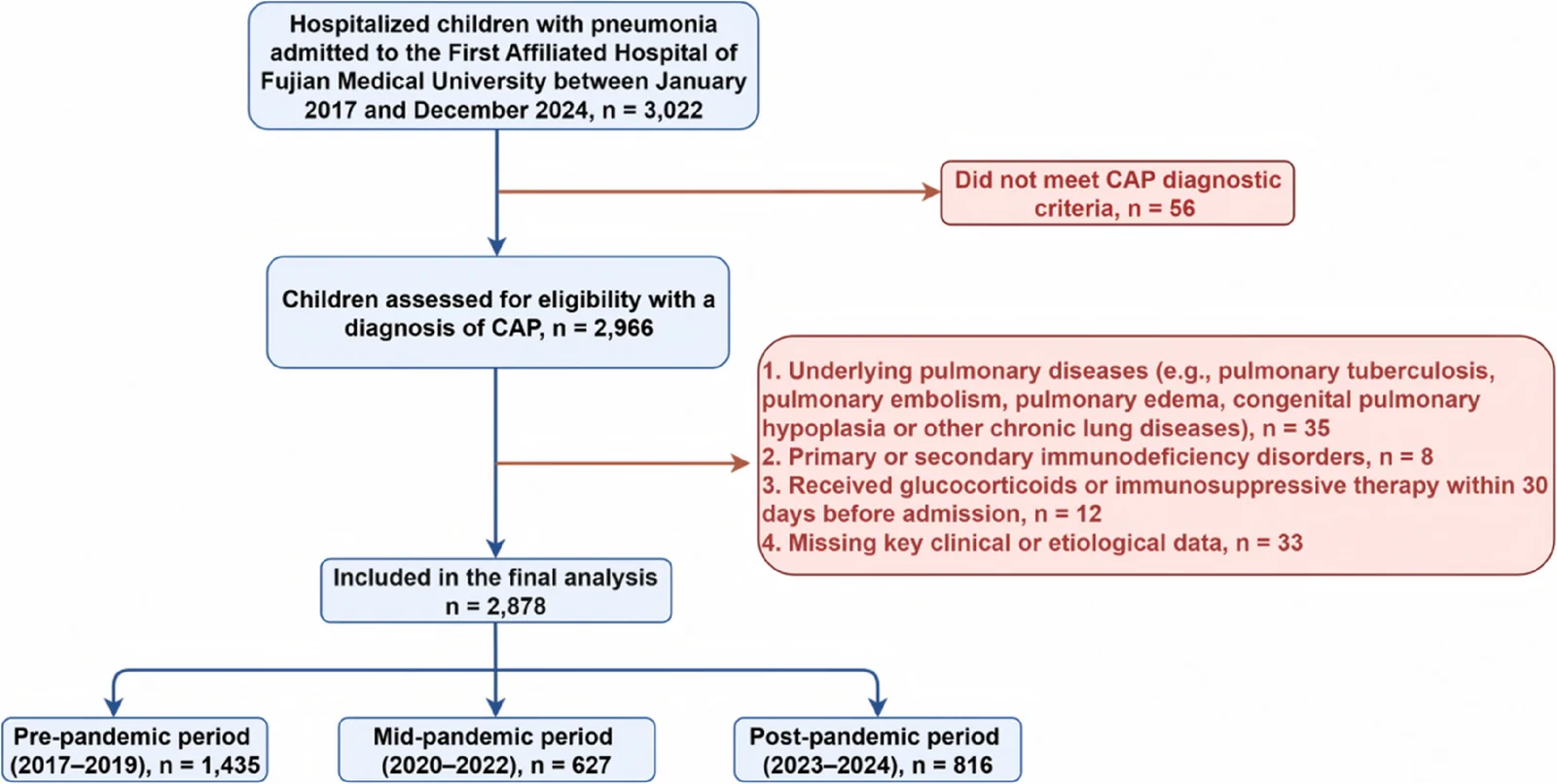
Flow chart of patient selection, exclusion, and enrollment

Exclusion criteria were as follows: ① underlying pulmonary diseases: any history of pulmonary tuberculosis, pulmonary embolism, pulmonary edema, congenital pulmonary hypoplasia or other underlying lung diseases (*n* = 35); ② immune system diseases: primary or secondary immunodeficiency diseases (*n* = 8); ③ recent immunosuppressive therapy: receipt of glucocorticoids or immunosuppressive therapy within 30 days prior to admission (*n* = 12); ④ missing key clinical or etiological data (*n* = 33). Additionally, in accordance with local public health policies during the pandemic (2020–2022), patients with confirmed or suspected SARS-CoV-2 infection were primarily referred to designated isolation facilities for treatment and were therefore effectively excluded from our inpatient cohort. After these exclusions, 2,878 children were included in the final analysis. The patient selection, exclusion, and enrollment process is shown in Fig. 1. Therefore, the children ultimately included in the analysis were generally free of significant underlying diseases that could substantially affect the respiratory pathogen spectrum or host immune status, which helped ensure the relative homogeneity of the study population.

This study has been approved by the Ethics Committee of the First Affiliated Hospital of Fujian Medical University (approval no. 2024 − 365). As this was a retrospective study, the Ethics Committee waived the requirement for informed consent from parents/guardians.

### Data collection

Demographic information (sex, age), clinical course data (date of admission, date of discharge, length of hospital stay), and laboratory test results were extracted from the hospital’s electronic medical record system (Hospital Information System, HIS). Only hospitalized pediatric patients with CAP were included in this study. To ensure methodological comparability across the study period (2017–2024), etiological testing for all enrolled cases was performed within 24 h of admission using standardized specimen collection procedures and a relatively consistent microbiological testing strategy. Specifically, specimen types were selected based on the target pathogen to ensure diagnostic specificity: deep respiratory specimens including induced sputum, intratracheal aspirates and bronchoalveolar lavage fluids were prioritized for the detection of *Streptococcus pneumoniae* (Spn), whereas pharyngeal swabs were utilized for the detection of viruses [influenza A virus (FluA), influenza B virus (FluB), RSV] and atypical bacteria [MP, *Chlamydophila pneumoniae* (Cpn), and *Legionella pneumophila* (Lp)]. Spn was detected by standardized bacterial culture using Columbia Blood Agar plates, with identification confirmed by Optochin susceptibility testing; MP was detected by Real-time PCR using the MP and Macrolide-Resistant Isolates Diagnostic Kit (Jiangsu Mole Bioscience Co., Ltd., Taizhou, China) targeting the MP P1 gene and 23 S rRNA A2063G/A2064G mutation sites, performed on the ABI 7500 Real-Time PCR System; Cpn, Lp, FluA, FluB, and RSV were detected using Chemiluminescent Immunoassay kits from Shenzhen New Industries Biomedical Engineering Co., Ltd. (SNIBE). The target pathogens of interest (MP, FluA, FluB, RSV, Spn, Cpn, and Lp) remained unchanged throughout the study period, and all tests were conducted by the Clinical Microbiology Laboratory of the First Affiliated Hospital of Fujian Medical University in accordance with its standard operating procedures. For each pathogen, the number of positive cases, occurrence of co-infections, and the proportion of severe pneumonia were recorded. Weight change was defined as the difference between body weight at discharge and body weight at admission (discharge weight − admission weight), with positive values indicating weight gain during hospitalization and negative values indicating weight loss. The relevant data were obtained from routine clinical weight measurements recorded during hospitalization.

The diagnosis of severe pneumonia was based on the Guideline for the Management of Community-Acquired Pneumonia in Children (2024 Revision) to ensure diagnostic consistency and accuracy [11]. According to this guideline, a case was classified as severe CAP if it met any one of the following criteria: poor general condition; refusal to eat or signs of dehydration; impaired consciousness; hypoxemia; involvement of multiple lobes or ≥ 2/3 of a single lobe; pleural effusion; extrapulmonary complications. This diagnostic definition was applied uniformly to all cases across the pre-pandemic, mid-pandemic, and post-pandemic periods.

### Statistical analysis

Data processing and statistical analysis were performed using R statistical software (version 4.3.1).

Categorical variables were presented as n (%), and intergroup comparisons were performed using the Pearson chi-square test. Continuous data were summarized as follows: for normally distributed variables, values were presented as mean ± standard deviation (SD); for non-normally distributed variables, values were presented as median (interquartile range, IQR; P25, P75). Intergroup comparisons of normally distributed continuous data were conducted using one-way ANOVA, whereas the Kruskal-Wallis H test was used for non-normally distributed continuous data.

Based on age-related growth and developmental characteristics, participants were classified into four age groups: infancy (0–1 year), early childhood (1–3 years), preschool age (3–6 years), and school age (6–14 years). Comparisons were then made across the pre-pandemic, mid-pandemic, and post-pandemic periods with respect to case distribution characteristics, pathogen-specific infection rates, and clinical outcomes. *P* < 0.05 was considered statistically significant.

## Results

### Analysis of baseline characteristics

A total of 2,878 children with CAP were included in the analysis. The overall mean (SD) age was 7.71 (3.13) years. Participants were stratified into three groups according to the pandemic phase: pre-pandemic (2017–2019, *n* = 1,435), mean (SD) age 8.79 (2.28) years; mid-pandemic (2020–2022, *n* = 627), mean (SD) age 5.91 (2.93) years; and post-pandemic (2023–2024, *n* = 816), mean (SD) age 6.52 (3.36) years.

Gender distribution: Of the 2,878 children, 1,668 (57.96%) were male and 1,210 (42.04%) female. No statistically significant difference was found in the sex ratio among the three pandemic stages (*P* = 0.642), indicating that sex was not a major risk factor for CAP incidence.

Age distribution: School-age children (6–14 years) were the predominant group among children with CAP; however, their proportion differed significantly across the three pandemic stages (*P* < 0.001), ranging from 90.73% in the pre-pandemic period to 40.03% in the mid-pandemic period and rising again to 54.78% in the post-pandemic period. During the mid-pandemic period, preschool-age children (3–6 years) formed the majority (45.30%). In the post-pandemic period, school-age children regained predominance, although the proportion of infants (0–1 year) also increased markedly compared with earlier stages (4.04%). These findings suggest increased susceptibility among younger children in the post-pandemic era.

Changes in body weight: There were significant differences in weight change categories among the three periods (*P* < 0.001). The proportion of children with weight gain in the post-pandemic period (27.57%) was significantly higher than that in the pre-pandemic (16.79%) and mid-pandemic periods (13.08%); in contrast, the proportion of those with weight loss was the highest in the mid-pandemic period (28.87%). See Table 1.

**Table 1:** Distribution and comparison of baseline characteristics among children with pneumonia in pre-pandemic, mid-pandemic, and post-pandemic periods

Variable	Total (*n* = 2,878)	Pre-pandemic (*n* = 1,435)	Mid-pandemic (*n* = 627)	Post-pandemic (*n* = 816)	*P*
Sex					0.642
Male, n (%)	1,668 (57.96)	837 (58.33)	369 (58.85)	462 (56.62)	
Female, n (%)	1,210 (42.04)	598 (41.67)	258 (41.15)	354 (43.38)	
Age, years [mean (SD)]	7.71 (3.13)	8.79 (2.28)	5.91 (2.93)	6.52 (3.36)	< 0.001
Age groups					< 0.001
Infant (0–1 year), n (%)	33 (1.15)	0 (0.00)	0 (0.00)	33 (4.04)	
Younger age (1–3 year), n (%)	198 (6.88)	0 (0.00%)	92 (14.67)	106 (12.99)	
Preschool children (3–6 year), n (%)	647 (22.48)	133 (9.27)	284 (45.30)	230 (28.19)	
School-age children (6–14 year)	2,000 (69.49)	1,302 (90.73)	251 (40.03)	447 (54.78)	
Weight changes					< 0.001
Gain, n (%)	548 (19.04)	241 (16.79)	82 (13.08)	225 (27.58)	
No change, n (%)	1,612 (56.01)	831 (57.91)	364 (58.05)	417 (51.10)	
Loss, n (%)	718 (24.95)	363 (25.30)	181 (28.87)	174 (21.32)	

data are presented as n (%) unless otherwise indicated

*yr* year, *SD* Standard deviation

*P* values were calculated using the Pearson chi-square test for categorical variables. The largest remainder method was applied to ensure the logical consistency of the data

### Trends in pathogen infection rates​

**Fig. 2:**
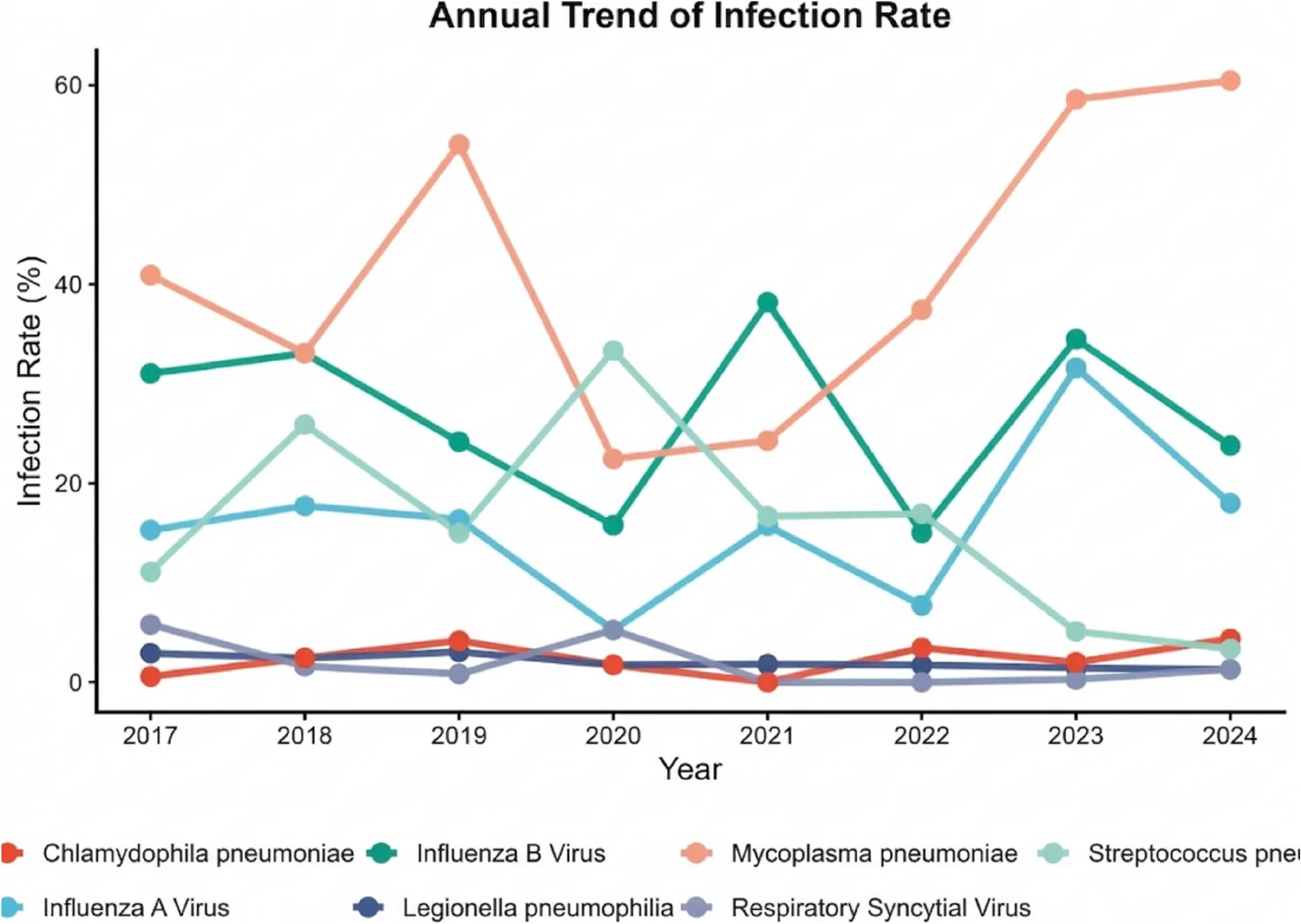
Trends in infection rates of predominant pathogens causing pediatric pneumonia from 2017 to 2024

As illustrated in Fig. 2, the line chart of pathogen infection rates shows that during the 2017–2024 study period, MP infection rates were low throughout the pandemic period (2020–2021) and then exhibited a linear increase from 2022 onward, reaching a single-year peak of 60.47% in 2024. This trend indicates a growing importance of MP in the etiological spectrum of pediatric pneumonia.

Flu rates fluctuated over time: a linear decline occurred from 2018 to 2020, followed by a rebound in 2021, and subsequent years showed alternating high and low levels. FluA and FluB displayed similar temporal patterns.

Spn infection rates showed a sustained decline beginning in the post-pandemic period of 2022. RSV exhibited a minor peak during the 2020 pandemic (5.26%) and remained at low levels thereafter. Cpn presented small peaks in 2019 (pre-pandemic, 4.17%) and 2022 (post-pandemic, 3.45%). Lp infection rates remained consistently low with no significant annual variation.

These findings indicate temporal variations in infection rates and pathogen distribution during the study period. See Fig. 2.

### Pathogen composition in each period​

The comprehensive analysis of 2,878 pediatric CAP cases revealed an overall pathogen positivity rate of 82.73%, defined as the proportion of cases with at least one pathogen detected (including both single infections and co-infections, with each case counted once). In the pre-pandemic, mid-pandemic, and post-pandemic periods, the positivity rates were 88.22%, 68.90%, and 83.71%, respectively, with a statistically significant difference (*P* < 0.001). The predominant pathogens identified were MP (36.31%), Flu (20.29%), and RSV (2.26%). See Table 2.

**Table 2:** Distribution of predominant pathogens in pediatric pneumonia across pre-pandemic, mid-pandemic, and post-pandemic periods

Variable	Total (*n* = 2,878)	Pre-pandemic (*n* = 1,435)	Mid-pandemic (*n* = 627)	Post-pandemic (*n* = 816)	*P*
Overall positivity rate, n (%)	2381 (82.73)	1266 (88.22)	432 (68.90)	683 (83.71)	< 0.001
MP, n (%)	1045 (36.31)	535 (37.28)	178 (28.38)	332 (40.69)	< 0.001
Flu, n (%)	584 (20.29)	381 (26.56)	115 (18.34)	88 (10.79)	< 0.001
RSV, n (%)	65 (2.26)	25 (1.73)	37 (5.90)	3 (0.37)	< 0.001
Spn, n (%)	54 (1.88)	25 (1.73)	12 (1.91)	17 (2.08)	0.508

data are presented as n (%)

*P* values were calculated using the Pearson chi-square test for categorical variables

### Temporal evolution of pathogen distribution

**Fig. 3:**
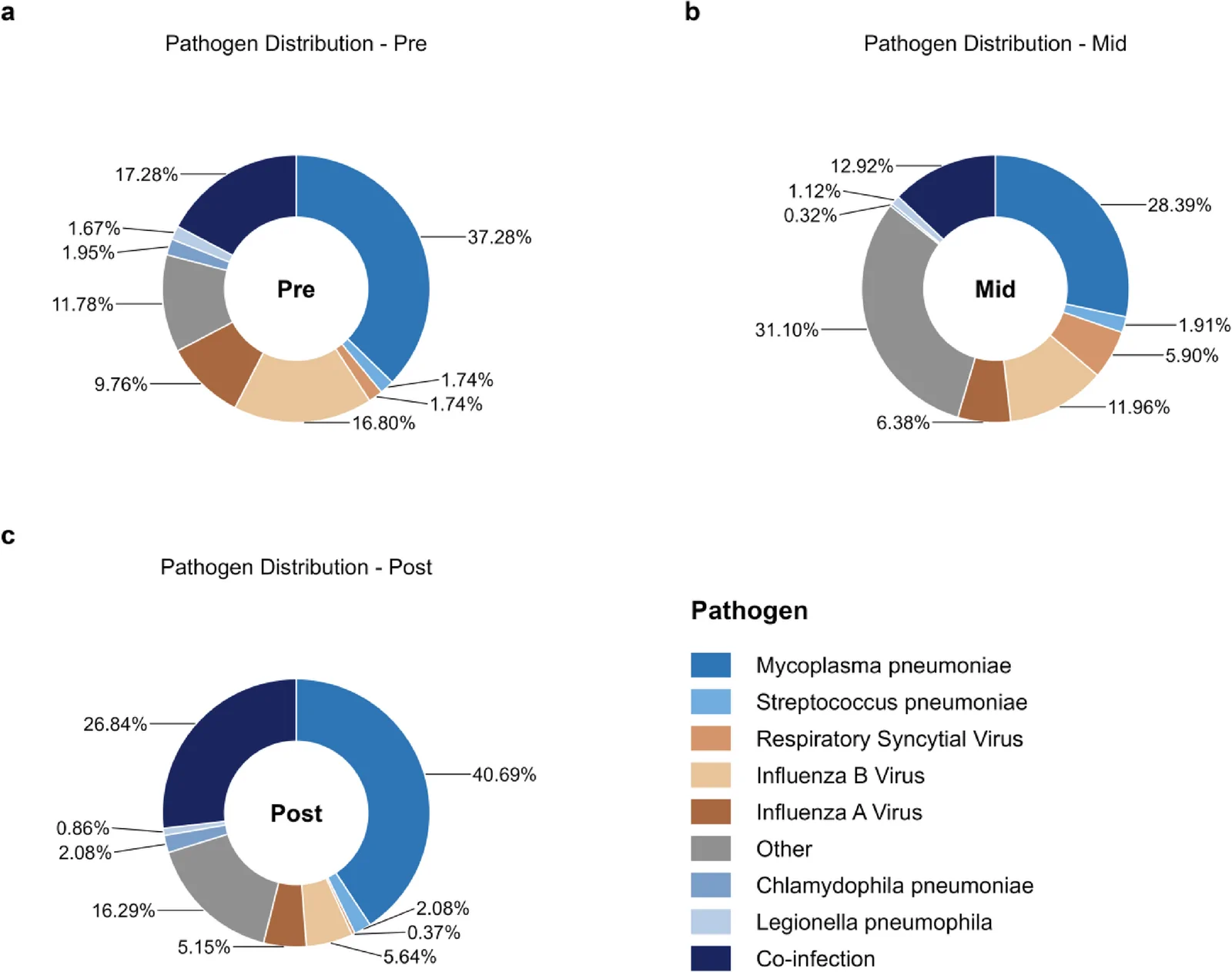
Distribution of pathogen categories among hospitalized children with community-acquired pneumonia across the pre-pandemic, mid-pandemic, and post-pandemic periods. (**a**) Pre-pandemic period (2017–2019); (**b**) mid-pandemic period (2020–2022); and (**c**) post-pandemic period (2023–2024)

Figure 3a–c shows the distribution of pathogen categories among hospitalized children with community-acquired pneumonia across the three periods.

#### Pre-pandemic period (2017–2019)

MP was the most prevalent pathogen, comprising 37.28% of cases, followed by FluB (16.80%) and FluA (9.76%), respectively. The remaining pathogens-Spn, RSV, Cpn, and Lp-each accounted for no more than 2.00%.

#### Mid-pandemic period (2020–2022)

The proportion of MP was significantly lower than in the pre-pandemic period (28.38%), although it remained the dominant pathogen. Among influenza viruses, FluA accounted for 6.38% and FluB for 11.96%, both lower than the corresponding pre-pandemic proportions. The proportion of co-infections decreased markedly to 12.92%, whereas the proportion of RSV increased to 5.90%.

#### Post-pandemic period (2023–2024)

The proportion of MP increased to 40.69%, remaining the predominant pathogen. Single-virus FluA 5.15%, FluB 5.64% were lower than in both the pre-pandemic and mid-pandemic periods, whereas co-infection rates involving MP increased significantly. The proportion of co-infections increased, whereas the proportion of RSV decreased to 0.37%. 

### Characteristics of co-infections in pediatric CAP

A total of 2,878 children with CAP were enrolled in this study, of whom 548 had co-infections, corresponding to an overall co-infection rate of 19.04%. The co-infection rates in the pre-pandemic, mid-pandemic, and post-pandemic periods were 17.28% (248/1,435), 12.92% (81/627), and 26.84% (219/816), respectively.

### Comparative analysis across pandemic periods​

In the pre-pandemic period, dual-pathogen patterns predominated, with the most frequent being FluB + MP (78 cases, 31.45%), followed by the triple-pathogen pattern FluB+FluA + MP (54 cases, 21.77%) and the dual-virus pattern FluA+FluB (36 cases, 14.52%). In the mid-pandemic period, FluB + MP remained the most common co-infection pattern (31 cases, 38.27%), followed by the triple-pathogen pattern FluB+FluA + MP, whose proportion (13.58%) was significantly lower than that in the pre-pandemic period (21.77%). In the post-pandemic period, the co-infection pattern shifted, with FluB+FluA + MP becoming the predominant type (83 cases, 37.90%). See Table 3.

**Table 3:** Comparison of major co-infection patterns and clinical characteristics across pandemic periods

Clinical Characteristics	FluB + MP	FluA+FluB	FluA + MP	FluB+FluA + MP
Positive Cases, *n* (%)				
Pre	78 (31.45)	36 (14.52)	20 (8.06)	54 (21.77)
Mid	31 (38.27)	9 (11.11)	5 (6.17)	11 (13.58)
Post	43 (19.63)	17 (7.76)	32 (14.61)	83 (37.90)
Mean Hospital Stay, days				
Pre	6.6	7.7	11.2	8.1
Mid	2.0	7.4	8.0	5.2
Post	6.8	5.6	6.3	5.4
Severe Cases, %				
Pre	11.5	11.1	35.0	16.7
Mid	16.1	11.1	20.0	27.3
Post	2.3	0	9.4	7.2

Pre, pre-pandemic period (2017–2019); Mid, mid-pandemic period (2020–2022); Post, post-pandemic period (2023–2024). Data on positive cases are presented as n (%)

Clinical features also varied across the study periods. In the pre-pandemic period, the longest mean hospital stay was observed in the FluA + MP group (11.2 days), which also had the highest proportion of severe cases (35.0%). In the mid-pandemic period, FluA + MP remained the pattern with the longest mean hospital stay (8.0 days), whereas FluB+FluA + MP showed the highest severe case proportion (27.3%). In the post-pandemic period, mean hospital stay ranged from 5.4 to 6.8 days across the major co-infection patterns, and the highest severe case proportion was observed in the FluA + MP group (9.4%), while no severe cases were observed in the FluA+FluB group.

## Discussion

CAP remains a major global public health challenge in pediatric populations. The complexity of its etiological spectrum and the dynamic nature of epidemiological patterns present persistent challenges for clinical management and public health strategies [7]. In this retrospective study, we analyzed 2,878 hospitalized children with CAP in Fujian Province from 2017 to 2024, systematically examining shifts in infection rates and pathogen profiles across the pre-pandemic, pandemic, and post-pandemic periods. These findings may contribute to a better understanding of temporal changes in pediatric pneumonia epidemiology in the post-COVID-19 era.

### Impact of the pandemic on infection rates and demographic characteristics

Our analysis revealed a V-shaped rebound in the overall pathogen positivity rate across the three pandemic phases (88.22% pre-pandemic→68.90% during the pandemic→83.71% post-pandemic). This pattern may be associated with the reduction in respiratory pathogen transmission during the period of non-pharmaceutical interventions (NPIs), followed by a rebound after relaxation of these measures [6, 12].

A noteworthy finding was the absence of hospitalized CAP cases in infants and young toddlers during the pre-pandemic and mid-pandemic phases (Table 1). Because this study included only hospitalized children, this pattern may have been influenced by multiple factors, including healthcare-seeking behavior, admission practices, and changes in exposure opportunities across age groups.

With respect to demographic characteristics, the proportion of cases in children under 1 year of age changed in the post-pandemic period [13]. This shift may be related to changes in exposure opportunities during the pandemic, including reduced social contact and altered healthcare-seeking behavior among younger children. Furthermore, children infected with SARS-CoV-2 typically experience mild illness, a phenomenon that has been reported in previous studies and may be related to differences in immune response profiles in children [14].

In addition, significant differences in in-hospital weight change categories were observed across the three periods. However, these findings should be interpreted cautiously. Because detailed nutritional and clinical data were unavailable in the present study, the observed differences in weight change patterns cannot be regarded as direct evidence of changes in nutritional status and should be considered descriptive and exploratory.

### Restructuring and rebound of the pathogenic spectrum

Our findings revealed a marked shift in predominant pathogens. Before the pandemic, MP and influenza viruses were the leading causes of pediatric CAP.

During the pandemic, NPIs curtailed the spread of MP and influenza viruses, while altering the relative proportions of other pathogens, such as RSV [15].

Following the relaxation of control measures, the post-pandemic period saw a rapid rebound in MP infection rates and co-infection rates, which became the predominant pattern, with MP reaching 40.69%. This resurgence may be partly related to the resumption of interpersonal contact after the relaxation of control measures, together with the known transmissibility characteristics of MP [12, 16].

Taken together, these observations support the importance of continued surveillance of common respiratory pathogens, particularly MP and influenza viruses, in pediatric CAP in the post-pandemic era.

### Co-infections and the risk of severe disease​

In this study, the co-infection rate rose markedly in the post-pandemic period (17.28% pre-pandemic→12.92% during the pandemic→26.84% post-pandemic), with MP+FluB and MP+FluA+FluB being the most frequent combinations across all phases. Children with co-infections tended to have a higher proportion of severe clinical features, such as persistent high fever and prolonged cough [17]. These features may reflect synergistic pathogenic interactions between co-occurring agents or the triggering of excessive inflammatory responses [18]. These observations suggest that co-infection patterns may be associated with differences in clinical severity, although the underlying mechanisms could not be determined from the present data [18, 19].

Accordingly, greater attention to co-infection patterns may be helpful in the clinical assessment of hospitalized children with CAP.

### Study limitations and prospects​

This study represents a single-center retrospective analysis and may be subject to geographical bias. Regarding SARS-CoV-2, data were not included in the present analysis. During the mid-pandemic period (2020–2022), patients with confirmed or suspected SARS-CoV-2 infection were isolated and treated in designated specialty hospitals according to policy, resulting in their natural exclusion from our inpatient cohort. In the post-pandemic period (2023–2024), SARS-CoV-2 testing was largely performed on an outpatient basis using self-purchased kits, leading to incomplete documentation of test results within the inpatient electronic medical record system.​ In addition, many patients with mild COVID-19 were managed in outpatient settings. Consequently, the reported co-infection patterns in this study reflect only conventional respiratory pathogens and should be interpreted with caution. In addition, the lack of data on COVID-19 vaccination status and pathogen antimicrobial resistance limits the ability to comprehensively assess the drivers of shifts in the etiological spectrum.

Furthermore, because detailed nutritional and comprehensive clinical data were unavailable, the observed differences in weight change categories could not be interpreted as direct indicators of nutritional status, and related findings should therefore be considered descriptive and exploratory. Moreover, common underlying conditions that may affect clinical outcomes, such as asthma, were not systematically adjusted for in the present analysis. Because this was a retrospective study and the relevant diagnostic information was not recorded in a fully standardized format in the electronic medical record system, residual confounding may have been introduced when interpreting certain clinical outcomes. Future research should adopt multicenter prospective designs and integrate multisource data-including clinical, epidemiological, and etiological information-to more thoroughly elucidate the epidemiological patterns and inform targeted prevention and control strategies for CAP in the post-pandemic era.

## Conclusion​

This single-center study observes temporal changes in the epidemiological profile of pediatric pneumonia in Fujian Province across the pre-pandemic, pandemic, and post-pandemic periods. These findings highlight the importance of continued respiratory pathogen surveillance and further investigation of co-infection patterns in hospitalized children with CAP in the post-pandemic era.

## Data Availability

The datasets used and/or analyzed during the current study are available from the corresponding author on reasonable request.
